# Advanced Digital Dentistry

**DOI:** 10.1155/2018/7540954

**Published:** 2018-12-26

**Authors:** Vahid Rakhshan, Chiarella Sforza, Predrag Vucinic, Anca M. Vitalariu, Márcio De Menezes

**Affiliations:** ^1^Department of Cognitive Neuroscience, Institute for Cognitive Science Studies, Tehran, Iran; ^2^Department of Dental Anatomy, Faculty of Dentistry, Islamic Azad University, Tehran, Iran; ^3^Department of Biomedical Sciences for Health, Università degli Studi di Milano, Milano, Italy; ^4^Department of Orthodontics, Clinic of Dentistry, Faculty of Medicine, University of Novi Sad, Novi Sad, Serbia; ^5^Department of Implantology, Removable Restorations and Technology, Universitatea de Medicina si Farmacie Grigore T. Popa Iasi, Iaşi, Romania; ^6^Department of Operative Dentistry, School of Health Sciences, State University of Amazonas, Manaus, Brazil

Advanced digital technology is rapidly changing the world, as well as transforming the dental profession. The adoption of digital technologies in dentistry allied with efficient processes, and accurate high-strength materials are replacing outdated techniques to improve overall patients' experiences and outcomes. A variety of digital devices such as laser scanners, holography, intraoral and face scanners, cone beam computed tomography (CBCT), software for computer-assisted design/computer-assisted manufacturing (CAD/CAM), and 3D printing provide new potential alternatives to replace the manual tasks and improve the quality of care and patient experiences. This technology has several advantages, including accurate measurements, storage, and time-saving as well as online consultation and presentation, providing information exchange with different centers for planning and appraising medical procedures and treatments. Even teaching and research tools greatly benefited from these innovations, with software for teaching dentistry (such as 3D software for learning anatomy or 3D simulation programs for surgical procedures), digital archiving of patient records and convenient/rapid sharing of them over the Internet, digital bibliographic assistance in dental research, or other utilities.

This special issue is a representative sampling of this literature on the use of digital technologies in dentistry. With the generous support of the respective editors, these papers cover an intentionally broad range of topics that include investigations of intraoral scanning accuracy, CBCT technology, 3D accuracy of digital impressions, 3D finite element models, and applications to orthodontics. Out of 25 manuscripts received in this issue, six were published: both technical aspects dealing with digital impressions (“Three-Dimensional Accuracy of Digital Impression versus Conventional Method: Effect of Implant Angulation and Connection Type” by M. Alikhasi et al. and “Trueness and Precision of Three-Dimensional Digitizing Intraoral Devices” by H. Mutwalli et al.) and imaging techniques (“Assessing the Correlation between Skeletal and Corresponding Soft-Tissue Equivalents to Determine the Relationship between CBCT Skeletal/Dental Dimensions and 3D Radiographic Soft-Tissue Equivalents” by D. I. Kim and M. O. Lagravere and “Accuracy of Periapical Radiography and CBCT in Endodontic Evaluation” by R. L. Giudice et al.) had been investigated. All these papers employed novel technologies to help answering clinical questions that may dictate different treatment plans.

Additionally, finite element studies (“Development and Validation of 3D Finite Element Models for Prediction of Orthodontic Tooth Movement” by U. Likitmongkolsakul et al.) and longitudinal assessments (“Three-Dimensional Changes of the Auditory Canal in a Three-Year Period during Adolescence Using CBCTs” by A. Woods and M. O. Lagravere) during growth and development have been performed. In both investigations, proper instruments and software were employed to offer quantitative information to the clinicians.

In synthesis, this special issue wants to give some examples of the current solutions that technology offers to dental practitioners and provide the bases for future investigations bringing essential ingredients for patient care, dental research, education, and daily practice.

